# Evaluation of Newly Designed and Traditional Punches in Manufacturing of Scored ODTs

**DOI:** 10.3390/pharmaceutics14102054

**Published:** 2022-09-27

**Authors:** Luca Palugan, Saliha Moutaharrik, Alessandra Maroni, Anastasia Anna Foppoli, Alice Melocchi, Carlo Vecchio, Andrea Gazzaniga, Matteo Cerea

**Affiliations:** Dipartimento di Scienze Farmaceutiche (DISFARM), Sezione di Tecnologia e Legislazione Farmaceutiche ‘M.E. Sangalli’ GazzaLaB, Università degli Studi di Milano, 20133 Milan, Italy

**Keywords:** orally disintegrating tablets, furosemide scored tablets, punch design, easy breakable tablets, quality by design, design of experiments, breakability panel test

## Abstract

To overcome difficulties in splitting, uneven breaking and inconsistent dosing frequently reported with scored tablets, a novel punch was proposed for the manufacturing of easy breakable tablets (EBTs). In this work, the performance of the EBT punch was investigated vs. a ridged one for traditional breakable tablets (TBTs) using a furosemide powder formulation for orally disintegrating tablets (ODTs). A Face Centered Central Composite Design was applied to investigate the influence of punch type, compaction force, tablet weight and press rotation speed on the mechanical properties of ODTs, their behavior in aqueous fluids and aptitude for splitting. Mass uniformity and adequate crushing strength, friability, water uptake, disintegration and wetting times were obtained from both TBTs and EBTs. Interestingly, more favorable splitting behavior was shown by tablets manufactured by the novel punch, in view of lower mass loss and portion mass variability after breaking. The ease of breaking, accuracy of subdivision and mass loss of ODTs were also evaluated by a volunteer (*n* = 20) panel test. Less difficulty was found in splitting EBTs than TBTs (p < 0.05), and a larger number of tablets were properly broken into four parts. Thus, this study proved the usefulness of the EBT punch in overcoming drawbacks associated with divisible tablets.

## 1. Introduction

In the last years, orally disintegrating tablets (ODTs) have attracted a great deal of attention as a dosage form suitable to increase the patient compliance. ODTs have been defined as solid dosage forms containing medicinal substances, which disintegrate rapidly, usually within a matter of seconds, when placed upon the tongue [1].

In addition to the advantages of solid oral dosage forms, due to their ability to disintegrate rapidly upon contact with the saliva, ODTs could address many patient needs, such as convenient administration for dysphagia sufferers, more commonly pediatric, geriatric, psychiatric and patients with nausea, vomiting and motion sickness complications [2]. Moreover, ODTs would facilitate dosing when there is no access to water, which often makes them the preferred alternative to conventional tablets and capsules [3]. Finally, they would allow for a prompt onset of action and, in some cases, drug absorption directly from the mouth avoiding or reducing first-pass metabolism.

ODTs can be obtained with different techniques, yielding dosage forms with different physical characteristics, especially in terms of mechanical properties [4,5]. Direct compression is the most advantageous because of the use of conventional equipment, extensive know-how and low costs [6]. However, since ODTs should be endowed with high porosity to improve water uptake and enable rapid disintegration, they commonly require to be tableted at low compaction forces. This may result in mechanical strength and friability issues, and thus in problems with their handling and the accuracy of the drug dose administered [3].

Scored ODTs would offer additional advantages in terms of dose flexibility and personalization, which may be reflected in a reduction in health care expenditure [7,8,9,10,11,12]. One or more score lines on the surface of tablets are useful to split them by hand into two, three or four portions. Otherwise, the use of a splitter, scissors or a knife could help divide the tablet into subunits.

The uneven breaking of tablets may result in inconsistent dosing and, consequently, in variable drug plasma levels that could be clinically relevant especially for drugs with a narrow therapeutic range.

The accuracy of subdivision needs to be evaluated by determining the uniformity of mass of the portions generated by splitting, according to the relevant monographs in the European Pharmacopoeia and United States Pharmacopeia. Moreover, an article in the Stimuli to Revision Process section of Pharmacopeial Forum, titled Pharmacopeial Standards for the Subdivision Characteristics of Scored Tablets, indicates that the ease of subdivision along the score lines should be considered as well as the loss of mass due to possible crumbling or detachment of small fragments associated with tablet splitting [13].

Many patients were reported to perceive scored tablets as having bad splitting behavior, which might consequently lead to non-compliance issues [14]. These negative feedbacks were mainly related to dysfunctional score lines, which lead to difficulties in subdivision. Moreover, excessive loss of mass and poor reproducibility of the portion mass after breaking were highlighted in a number of studies [12,14]. Characteristics of the tablets, such as the size, shape, hardness and presence as well as geometry of the score lines were shown to have a strong impact on the degree of difficulty encountered in breaking a tablet [11,12]. In this respect, continued improvement in the design of punches is necessary.

Recently, a newly designed ridged punch was proposed for the manufacturing of cross-scored easy breakable tablets (EBTs) [15]. The novel EBT punch was successfully employed for the preparation, starting from a number of co-processed excipients of *placebo* ODTs of 8 mm in diameter, which showed satisfactory mechanical properties on the one hand, and adequate water uptake as well as disintegration performance on the other.

Based on the above premises, the aim of the work was to study the performance of the novel EBT punch for the manufacturing of furosemide-containing cross-scored ODTs and to compare it with a traditional cross-scored punch (TBT).

Furosemide is a loop diuretic, used primarily for the treatment of edema in congestive heart failure, belonging to Class IV under the Biopharmaceutics Classification System (BCS) having low solubility and permeability [16,17]. It is a weakly acidic drug, mostly absorbed in the stomach and upper intestine. Moreover, it can be absorbed in the buccal cavity. For this reason, an ODT formulation could enhance its bioavailability. Since the furosemide dose is regularly adapted to the age and conditions of patients, scored tablets would provide flexibility in dosing, possibly reducing the number of product strengths on the market and overcoming stability problems related to the use of liquid formulations (e.g., suspensions and solutions).

Furosemide-containing ODTs obtained under different tableting conditions were evaluated for overall quality, with special focus on splitability. Due to the number of factors that can have an impact, Quality by Design was applied by using the Design of Experiments (DoE) methodology [18]. Moreover, a volunteer panel test was set to explore the ease of breaking as perceived by end users, the accuracy of subdivision of tablets and the relevant mass loss.

## 2. Materials and Methods

### 2.1. Materials

Furosemide was kindly donated by Teknofarma (Torino, Italy), as was Prosolv^®^ ODT G_2_ by JRS Pharma (Rettenmaier Italia, Castenedolo, Italy). Magnesium stearate was supplied by ACEF (Fiorenzuola d’Arda, Italy) and colloidal silica (Aerosil^®^ 200) by Evonik (Essen, Germany).

### 2.2. Manufacturing and Characterization of Tablets

Powder blends (197 g) composed of furosemide, Prosolv^®^ ODT G_2_ and colloidal silica were obtained in a Turbula^®^ mixer (Willy A. Bachofen, Muttenz, CH; 15 min, 200 rpm) (Table 1). Afterwards, magnesium stearate (3 g) was added and mixed for 3 min.

The bulk density of the powders was measured using a 250 mL glass cylinder (about a 100 g sample), and the compressibility index was calculated (STAV 2003, Jel, Ludwigshafen, Germany).

Different amounts of powder mixture (160, 200 and 240 mg) were tableted by a rotary instrumented press (AM8S, Officine Meccaniche Ronchi, Cinisello Balsamo, Italy) equipped with Ø 8 mm easy breakable tablet (EBT) or traditional breakable tablet (TBT) punches (B&D Italia, Carate Brianza, Italy) and a force feeder system. The ODTs were manufactured under different levels of compaction force (Fa) and rotation speed (RS) (Table 2). Tablet mass uniformity was assessed by calculating the coefficient of mass variation (CV_T_) of 20 units (analytical balance Europe 500, Gibertini, Novate Milanese, Italy). Diametral crushing strength (Fcd) was measured by placing a single tablet horizontally between the jaws of the crushing tester with the non-indented face laid on the stainless-steel surface and one score line oriented in the same direction as the applied force (TBH-28, Erweka, Langen, Germany; *n* = 10) [15]. Axial crushing strength (Fca) was measured with the same tester by placing a single tablet vertically between the jaws, with its scored face leaning against the static jaw of the equipment so that a stable position of the unit could be maintained during the test (*n* = 10).

Friability (Fri) (friabilometer TA3, Erweka, Langen, Germany) and disintegration time (DT) (disintegration apparatus DT3, Sotax, Aesch, Switzerland) were measured according to the European Pharmacopoeia. Wetting time (WT) was assessed by visual detection as the time needed to dissolve approximately 2 mg of methylene blue added to the upper surface of a tablet placed on a wet paper tissue in a Petri dish (11 cm diameter, 15 g water, *n* = 3) [19]. Percentage water uptake (PWU) was calculated as the percentage weight gain of a tablet placed on a wet paper tissue in a Petri dish until complete imbibition (11 cm diameter, 25 g water, *n* = 3).

Mass variability of portions (CV_P*ct*_) was calculated as the CV of the mass of the parts obtained from axial breaking of the tablet performed by crushing tester (*n* = 30 tablets).

Mass loss upon breaking (ML*_ct_*) was calculated as the percentage ratio between the difference in the mass of the intact tablet and the sum of the relevant subdivided portions and the mass of the intact tablet (*n* = 30) [20].

### 2.3. Breakability Panel Test

Twenty adults of different ages (23–79 years), male (8) and female (12), left (3) and right (17) dominant hand were enrolled for the test. They were asked to break the cross-scored tablets by applying force with the index finger of their dominant hand onto the units placed with the scored face upward on a flat and hard surface. Four batches were selected, and 3 tablets for each batch were tested.

The difficulty experienced in breaking the tablet was quantified by the volunteers (DB) by assigning a score on a ten-point scale for each of the tested tablets, where score 0 indicated no difficulty and score 10 corresponded to unfeasible splitting. The percentages of tablets that were successfully divided into 4 parts (TB4) and that were not divided at all (TB0) were recorded. In the case of tablets that were successfully divided into 4 parts, mass loss after breaking (ML*_pt_*) and mass variability (CV_P*pt*_), calculated on all the resulting portions, were also assessed.

### 2.4. Statistical Analysis

The Design of Experiments setting, multiple regression of characterization results and calculation of the significance of the regression models were performed using MiniTab software (Minitab 18, GMSL, Nerviano, Italy). Results from the panel test were elaborated with Excel Microsoft 365 (Microsoft, Richmond, WA, USA).

## 3. Results

**Figure 1:**
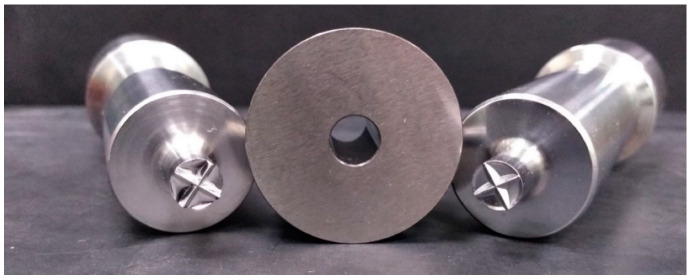
EBT (**left**) and TBT (**right**) upper punches with the relevant die.

**Figure 2:**
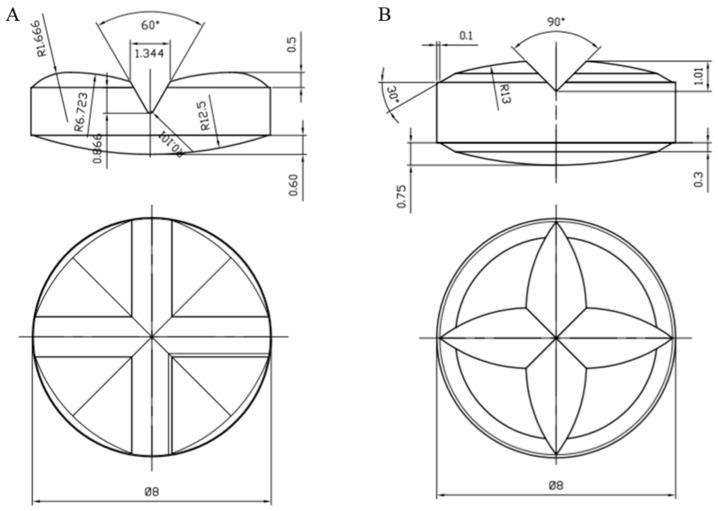
Technical drawings of scored tablets (side and top views) from EBT (**A**) and TBT (**B**) punches (dimensions are expressed in mm).

A comparative evaluation of the ridged punch proposed for the manufacturing of cross-scored EBTs was carried out vs. a traditional one. Photographs of the upper EBT and TBT punches with common cylindrical die and technical drawings of the relevant scored tablets are presented in Figure 1 and Figure 2, respectively. The novel punch exhibited a peculiar geometry of the upper element that was purposely designed to include a more pronounced cross ridge and a double curvature. The tablets as manufactured by the EBT punch were conceived to present, as compared to the traditional breakable ones, (i) a smaller section area of fracture resulting from deeper score lines and (ii) an increased distance between the points of application of effort because of the double radius of the curvature. Particularly, the depth of the score lines was approximately 35% greater than in the TBTs obtained by a conventional ridged punch, employed as a reference, and the lever arms, i.e., the distance between the score line and the highest point of the tablet, were threefold longer. Such characteristics would allow a reduced amount of force to be applied with a fingertip for splitting. A deeper score line could also decrease the mass variability of the portions [12]. On the other hand, this would cause a greater compaction pressure to be exerted in the region of the scores, which may hinder tablet breakability by counteracting the benefits arising from the reduced area of fracture. Therefore, an appropriate balance between these opposite effects of a deeper indentation would be critical to the attainment of divisible tablets having the desired quality. The lower elements of both the EBT and TBT punch sets have a traditional design, with a single curvature radius and no ridges.

An evaluation of the performance of the novel and the traditional punches was carried out by applying a DoE and through an assessment of the breakability of the furosemide ODTs by a volunteer panel test.

### 3.1. DoE Evaluation of EBT and TBT Punches

Based on previous work demonstrating the suitability of the EBT punch for the manufacturing of divisible orally disintegrating *placebo* tablets, Prosolv^®^ ODT G_2_ was selected as the main component of the furosemide-containing formulation [15]. A glidant was included in order to improve the powder flow properties. In this respect, colloidal silica proved to be effective at 1.25% *w*/*w*, leading to a compressibility index close to 25%. Nonetheless, the use of a force feeder was necessary to enable proper filling of the dies during tableting. The mixture also included 1.5% *w*/*w* of magnesium stearate.

The influence of several processing conditions on the properties of the tablets prepared by the two punches was evaluated through DoE. The factors that were in principle identified as key parameters were: (i) compaction force (Fa), as it was expected to have an impact on crushing strength and, consequently, on tablet splitability, (ii) tablet mass (TW), which affects the section fracture area and, therefore, the splitting force, and (iii) press rotation speed (RS), which the mass uniformity and tablet hardness may depend on. Preliminary tests were conducted with the EBT punch to define the ranges and levels of the factors to be used in the study. Compaction force values around 6.0 kN turned out to be suitable for achieving proper mechanical characteristics on the one hand, and porosity along with disintegration properties of the tablets on the other. The range for this factor was thus set at ±20% of 6.0 kN.

Because tablets of 200 mg previously obtained by the EBT punch were proved to be suitable for handling, a 160–240 mg weight range was explored to study the effect of the mass of powder to be compacted [15]. In order to maintain 25 mg of furosemide dose and to keep the percentages of silica as well as magnesium stearate constant in the composition of all batches, the amount of Prosolv^®^ ODT G_2_ was adjusted accordingly. The resulting formulations are shown in Table 1. Finally, the press rotation speed was set at either 10 or 20 rpm, which represent the most common and maximum turret speeds with the tablet press in use, respectively. Since the compaction force and tablet weight were expected to be the most important factors impacting breakability, these were fixed at three levels. For each factor, the edges of the interval were subsequently transformed to −1 and +1 in normalized scales. The central values were set to 0. The factor levels relevant to the applied DoE are listed in Table 2.

**Table 2:** Levels of DoE factors.

		Levels		
*n*°	Factor	−1	0	+1
1	Compaction force, Fa (kN)	4.8	6.0	7.2
2	Tablet weight, TW (mg)	160	200	240
3	Press rotation speed, RS (rpm)	10	-	20
4	Punch type (PT)	EBT	-	TBT

Due to the number of factors and levels involved, a Central Composite Design was preferred over a Full Factorial Design in order to reduce the number of experimental trials from 36 to 24. Particularly, a Face Centered Central Composite Design (FCCCD) was carried out, so that a maximum of three instead of five levels could be set in the case of those factors for which quadratic effects were to be calculated. The final matrix of the experiments is shown in Table 3, where the levels of factors are listed for each batch to be manufactured.

The trials were run in random order. No critical issues were found in manufacturing, thus indicating the suitability of the experimental space selected for the study. Despite the complexity of the upper punch geometry, no major problems of powder adhesion were encountered in the tableting process.

As the responses (dependent variables), different characteristics were considered useful for describing the mass uniformity of the tablets, their mechanical properties, interaction with fluids and accuracy of subdivision. In particular, tablets were characterized in terms of mass variability (CV_T_), diametral and axial crushing strength (Fcd and Fca), friability (Fri), wetting time (WT), percentage water uptake (PWU) and disintegration time (DT). After each unit was broken into four portions by the crushing tester, the mass lost as powdered/crumbled material or small fragments (ML*_ct_*) and the mass variability of the subdivided portions (CV_P*ct*_) were assessed.

Results from the characterization of the tablet batches manufactured according to the FCCCD are listed in Table 4.

ODTs with reproducible mass and mechanical strength were obtained under all combinations of experimental conditions provided by the DoE. The units were shown to take up water from 13 to 46% of their weight in a few seconds and to disintegrate within 3 min in the majority of cases, never exceeding 6 min. Mass loss upon subdivision was always <4%, and weight variability of the portions obtained after splitting, as expressed by the coefficient of variation, ranged from 7 to 28%.

The data collected were processed to calculate the coefficients of the mathematical model describing the relationship between each response and all factors considered in the study (Equation (1)).

y = b_0_ + Σb_i_x_i_ + Σb_ij_x_i_x_j_ + Σb_ii_x_i_^2^ (1)

where:

y is the value of the response (dependent variable) under evaluation;

x_i_ is the level of the factors (independent variables) involved in the study (1 = Fa, 2 = TW, 3 = RS and 4 = PT);

b_0_ is the intercept;

b_i_ is the linear coefficient of the i^th^ factor (where i indicates the 1–4 factors);

b_ij_ is the coefficient of the interaction between i^th^ and j^th^ factors;

b_ii_ is the quadratic coefficient of the i^th^ factor.

Calculations of the above-mentioned coefficients were performed by multiple linear stepwise regression using the least squares method. Stepwise regression was applied to maximize the R^2^_adj_ value. The regression coefficients along with the correlation coefficients are shown in Table 5.

The equation describing CV_T_ confirmed that the tablets from all the batches had an overall reproducible weight, as highlighted by the low value of the b_0_ coefficient, regardless of the punch type (PT) employed. Furthermore, this response turned out to be influenced by press rotation speed only, although to a very low extent, thus demonstrating the benefits of using the force feeder.

With regard to the mechanical properties, as expected, compaction force and tablet weight exerted positive and synergistic effects on both diametral and axial crushing strengths, whereas punch type seemed to impact friability only. The values obtained for this response were in any case acceptable, being well below the limit of 1%. So, the effect of punch type could be considered practically irrelevant. With respect to the interaction with fluids, which is typically dependent on the tablet porosity, the mathematical models indicated that an increase in compaction force and tablet weight was reflected in increased wetting and disintegration times and decreased percentage water uptake. Particularly, the effects of these factors on wetting time were synergistic. The opposite effects of compaction force and tablet weight on the mechanical properties on the one hand, and the performance in aqueous medium on the other, lay emphasis on the need for proper setting of the tableting conditions in order to achieve dosage units that could bear handling while rapidly disintegrating upon contact with biological fluids.

Punch type did not impact the ability of tablets to interact with aqueous fluids. A slight influence was only found in the case of disintegration time. Finally, press rotation speed showed a negative effect on disintegration time and wetting time, reasonably due to a more porous and weaker structure resulting from a shorter dwell time.

Based on the relevant responses, the EBT punch exhibited a better performance in terms of splitability compared with the TBT one. Indeed, the tablets manufactured by this punch showed both a lower mass loss and mass variability of the portions generated by breaking. Compaction force and press rotation speed also influenced the reliability of tablet subdivision, in a negative and positive way, respectively. Accordingly, it would be advantageous to carry out the tableting process at a relatively high compaction force and low press rotation speed.

Interestingly, the negative coefficients of interaction between the compaction force and punch type (b14) obtained for the splitability responses, i.e., loss of mass and CVPct, indicated that increasing the compaction force would diminish the effect of punch type, thus resulting in more aligned values of such responses for the tablets manufactured by the EBT and TBT punches. Based on the overall results of the DoE, it was possible to obtain furosemide-containing scored ODTs with adequate mechanical characteristics and performance in the aqueous fluids via the use of both the TBT and EBT punches. Importantly, the peculiar geometry of the new punch allowed for a more favorable behavior on splitting with the formulation in use.

### 3.2. Panel Test Evaluation of EBT and TBT Punches

In order to evaluate the extent of the difficulty perceived by potential end users in breaking these ODTs, the comparative study was broadened to include a survey conducted with a panel test of volunteers.

The scored tablets previously manufactured within the DoE study were used for the panel test. The number of batches to be evaluated was set to four (two for each type of punch), and each of the 20 panelists involved were requested to split a limited number of tablets (three per batch for a total of 12) in order to reduce the risk of a non-objective judgment.

The four batches were selected based on the outcome of the FCCCD, particularly taking mass loss on breaking and accuracy of subdivision into account. Moreover, following preliminary trials of manual splitting, batches of tablets that turned out too hard were ruled out. Batches #1, #9, #21 and #24 were ultimately subjected to the test, i.e., those manufactured at the low or medium levels of compaction force (4.8 or 6.0 kN) and mass (160 or 200 mg), under low press rotation speed (10 rpm).

Volunteers were trained to break the tablets by applying force with the index finger of their dominant hand onto the unit placed on a hard flat surface with the scored face upward. They were asked to indicate the extent of difficulty experienced in subdividing the tablet (DS) by assigning a score on a ten-point scale for each tested unit, where 0 and 10 corresponded to no difficulty and unfeasible breaking, respectively.

**Figure 3:**
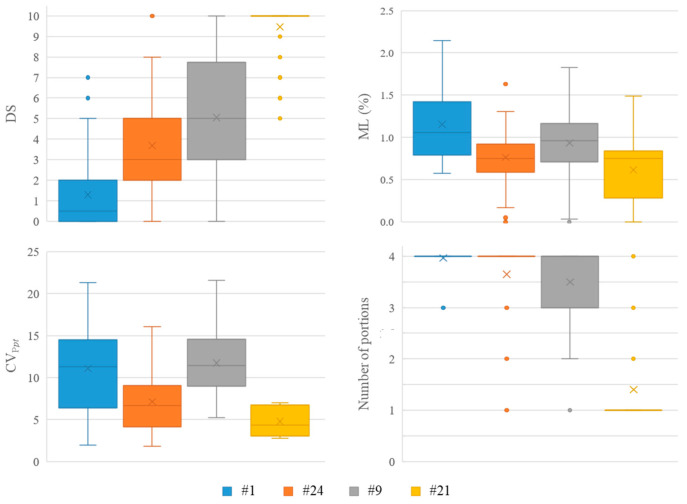
Box-and-whisker plots of the distribution of breakability panel test results (20 subjects, *n* = 3). DS, difficulty in tablet subdivision; ML*_pt_*, mass loss following splitting into four portions; CV_P_*_pt_*, coefficient of variation of portion mass; Number of portions, number of portions that were obtained after tablet manual splitting.

Results from the breakability panel test, expressed as the difficulty in tablet subdivision, the percentage of tablets that were successfully divided into four parts (TB4) and that of tablets that were not split at all (TB0), the mass loss after breaking (ML*_pt_*) and the mass variability of the resulting portions (CV_P*pt*_), are reported in Table 6. In Figure 3, the distributions of data relevant to difficulty in tablet subdivision, mass loss, CV_P*pt*_ and number of portions obtained by manual splitting are presented as box-and-whisker plots. Each box is delimited by the first and third quartile; the line inside the boxes and the cross indicate the median and arithmetic mean, respectively. Finally, the whiskers define the range of data excluding outliers, which are indicated by circles.

The ease of the subdivision of the ODTs manufactured under the same conditions by the EBT and TBT punches was clearly distinguished by the volunteers participating in the test. In particular, a lower mean difficulty in subdivision was assigned to EBTs (p < 0.05). This better score was clearly associated with a lower incidence of failure to break the tablets and greater success in properly splitting the units into four parts. Such results pointed out easier splitting of the ODTs manufactured by the novel punch, irrespective of the age, sex and dominant hand of the panelists (data not shown).

When the compaction force and tablet weight were increased, as expected, the subdivision of both EBTs and TBTs turned out to be more difficult. Notably, the volunteers generally managed to split the ODTs of Batch #1, manufactured by the EBT punch at the lowest compaction force and tablet weight, and four portions were recovered in a high percentage of cases.

The amount of material lost upon breaking was generally small (approximately 1%). The mass variability of the portions appeared to be more affected by the operating conditions than by the punch type. Indeed, with both EBT and TBT punches, the batches obtained at higher compaction force and tablet weight exhibited a lower weight variability of the portions derived from tablet splitting (p < 0.05). This indicated that increasing the tablet hardness would be reflected in higher accuracy of subdivision regardless of the greater splitting difficulties brought about.

Based on the results discussed above, the benefits associated with the use of the EBT punch were highlighted by the breakability panel test. Overall, the enrolled volunteers rated the novel punch superior as compared with the TBT one in terms of ease of splitting. Improved accuracy of subdivision was also assessed.

## 4. Conclusions

A newly designed punch for EBTs was compared with a traditional ridged one. Particularly, the relevant performance in the manufacturing of furosemide-containing ODTs was explored by evaluating mechanical and subdivision properties as well as behavior in aqueous fluids of the obtained tablets. For this purpose, an FCCCD was applied to test the punches in a relatively broad experimental space, and a breakability panel test was carried out recruiting 20 volunteers of different ages, sexes and dominant hands.

The results provided by the FCCCD indicated, for both the TBT and EBT punches, statistically significant effects of compaction force, tablet weight and press rotation speed on the overall characteristics of the scored ODTs. The EBT punch allowed tablets with adequate quality to be manufactured, showing advantages in breakability as assessed by the crushing test.

The ease of splitting found with the EBTs was strengthened by the panel test, which was able to discriminate between the two punches under investigation. Particularly, EBTs were judged by the volunteers as preferable over traditional scored ODTs, and the accuracy of subdivision was subsequently demonstrated.

On the basis of the overall results, the EBT punch was shown to be effective in tackling well-known issues related to scored tablets, even with a challenging ODT formulation, and considered worthy of further investigation using a range of powder blends. Particularly, it would be of interest to broaden the study of the novel punch to the manufacturing of breakable tablets that are usually obtained under compaction forces greater than with ODTs. A fine tuning of the operating conditions would allow a proper balance of patient convenience, handling and biopharmaceutical characteristics of the scored tablets to be achieved.

## Figures and Tables

**Table 1:** Composition of ODTs of different mass.

Component	Amount (mg)		
Furosemide	25.0	25.0	25.0
Prosolv^®^ ODT G_2_	130.6	169.5	208.4
Colloidal silica	2.0	2.5	3.0
Magnesium stearate	2.4	3.0	3.6
Total mass	160.0	200.0	240.0

**Table 3:** FCCCD matrix.

Trial	Coded Levels			
	Compaction Force (Fa)	Tablet Weight (TW)	Press Rotation Speed (RS)	Punch Type (PT)
1	−1	−1	−1	−1
2	+1	−1	−1	−1
3	−1	+1	−1	−1
4	+1	+1	−1	−1
5	−1	−1	+1	−1
6	+1	−1	+1	−1
7	−1	+1	+1	−1
8	+1	+1	+1	−1
9	−1	−1	−1	+1
10	+1	−1	−1	+1
11	−1	+1	−1	+1
12	+1	+1	−1	+1
13	−1	−1	+1	+1
14	+1	−1	+1	+1
15	−1	+1	+1	+1
16	+1	+1	+1	+1
17	−1	0	−1	+1
18	+1	0	+1	−1
19	0	−1	+1	−1
20	0	+1	−1	+1
21	0	0	−1	+1
22	0	0	+1	−1
23	0	0	+1	+1
24	0	0	−1	−1

**Table 4:** Characteristics of ODTs manufactured according to FCCCD. CV_T_, coefficient of variation of tablet mass; Fcd, diametral crushing strength; Fca, axial crushing strength; Fri, tablet friability; WT, wetting time; PWU, percentage water uptake; DT, tablet disintegration time; ML*_ct_*, mass loss following splitting into four portions; CV_P*ct*_, coefficient of variation of portion mass.

Batch #	CV_T_	Fcd^•^ (N)	Fca^•^ (N)	Fri^•^ (%)	WT^•^ (s)	PWU^•^ (%)	DT^•^ (min)	ML^•^ (%)	CV_P*ct*_
1	1.09	48 ± 5	37 ± 5	0.485	40 ± 4	30.1 ± 4.8	1.40 ± 0.04	0.36 ± 0.29	8.32
2	1.30	65 ± 7	46 ± 4	0.311	80 ± 25	31.3 ± 15.5	3.03 ± 0.40	0.38 ± 0.28	7.85
3	1.17	67 ± 8	68 ± 10	0.630	53 ± 8	35.9 ± 16.8	1.96 ± 0.35	0.78 ± 0.42	9.60
4	1.59	138 ± 9	160 ± 17	0.362	150 ± 46	21.9 ± 9.9	5.13 ± 0.07	0.19 ± 0.11	7.65
5	1.30	38 ± 7	21 ± 4	1.049	21 ± 1	37.7 ± 14.0	0.18 ± 0.25	1.01 ± 0.49	14.74
6	1.86	64 ± 7	38 ± 5	0.065	54 ± 28	24.9 ± 3.7	3.16 ± 0.15	0.29 ± 0.29	12.69
7	1.62	73 ± 6	80 ± 11	0.666	26 ± 15	27.2 ± 17.1	2.21 ± 0.24	0.88 ± 0.56	9.98
8	1.10	84 ± 20	108 ± 13	0.343	126 ± 3	32.9 ± 10.2	3.78 ± 0.35	0.29 ± 0.12	11.05
9	0.96	36 ± 8	37 ± 7	0.368	41 ± 12	46.0 ± 16.5	2.15 ± 0.13	2.33 ± 2.82	17.08
10	0.89	54 ± 9	61 ± 9	0.187	67 ± 18	27.5 ± 3.1	3.06 ± 0.05	0.57 ± 0.35	17.85
11	1.35	49 ± 13	69 ± 12	0.341	37 ± 28	40.3 ± 5.8	2.39 ± 0.14	1.44 ± 1.09	18.84
12	1.36	124 ± 13	155 ± 17	0.041	135 ± 23	14.6 ± 1.1	5.43 ± 0.04	0.42 ± 0.33	6.35
13	1.29	42 ± 8	36 ± 5	0.551	25 ± 9	45.6 ± 20.8	1.67 ± 0.42	3.90 ± 5.53	23.40
14	1.06	52 ± 7	56 ± 4	0.285	40 ± 10	17.9 ± 1.7	2.45 ± 0.07	0.75 ± 0.36	12.82
15	1.81	63 ± 11	71 ± 16	0.395	43 ± 10	19.8 ± 10.4	2.70 ± 0.34	2.62 ± 3.07	28.07
16	1.35	107 ± 18	139 ± 21	0.247	53 ± 5	13.8 ± 6.4	5.36 ± 0.18	0.52 ± 0.32	9.56
17	1.97	59 ± 11	73 ± 8	0.253	29 ± 10	17.8 ± 6.1	2.17 ± 0.54	1.09 ± 0.60	15.78
18	1.28	94 ± 16	87 ± 8	0.317	69 ± 17	39.6 ± 20.9	2.90 ± 0.52	0.10 ± 0.41	10.99
19	1.91	53 ± 13	41 ± 10	0.351	41 ± 6	29.8 ± 18.1	1.42 ± 0.04	0.57 ± 0.43	16.60
20	0.82	121 ± 9	150 ± 10	0.164	76 ± 18	18.5 ± 13.9	5.51 ± 0.47	0.50 ± 0.51	11.84
21	0.80	67 ± 7	72 ± 9	0.304	53 ± 12	22.9 ± 2.8	3.04 ± 0.50	0.79 ± 0.41	14.66
22	2.10	78 ± 8	76 ± 9	0.376	64 ± 4	27.5 ± 7.3	3.09 ± 0.44	0.71 ± 0.87	11.89
23	1.56	99 ± 19	99 ± 11	0.049	62 ± 37	22.4 ± 4.2	3.32 ± 0.24	1.11 ± 0.64	15.13
24	0.78	97 ± 13	89 ± 8	0.220	42 ± 15	35.0 ± 4.0	3.61 ± 0.38	0.41 ± 0.22	8.79

^•^ (mean ± sd).

**Table 5:** Regression coefficients of mathematical models (Equation (1)) of each response and relevant correlation coefficients. CV_T_, coefficient of variation of tablet mass; Fcd, diametral crushing strength; Fca, axial crushing strength; Fri, tablet friability; WT, wetting time; PWU, percentage water uptake; DT, tablet disintegration time; ML*_ct_*, mass loss following splitting into four portions; CV_P*ct*_, coefficient of variation of portion mass.

Coefficient	CV_T_	Fcd (N)	Fca (N)	Fri (%)	WT (s)	PWU (%)	DT (min)	ML*_ct_* (%)	CV_P*ct*_
b_0_	1.347 *	87.2 *	87.8 *	0.244 *	60 *	28.33 *	3.33 *	0.697 *	13.31 *
b_1_		17.2 *	21.0 *	−0.158 *	26 *	−4.54 *	1.03 *	−0.586 *	−2.63 *
b_2_		20.6 *	33.7 *		16 *	−3.34	0.83 *	−0.159	−1.11
b_3_	0.173 *	−3.3	−5.0	0.041	−9 *		−0.25 *	0.252 *	1.93 *
b_4_		−1.9	5.0	−0.089 *	5	−2.88	0.28 *	0.426 *	2.75 *
b_11_		−16.9 *	−13.3 *	0.151 *			−0.49		
b_22_								0.328	
b_12_		8.1 *	12.7 *		12 *		0.26		−1.22
b_13_				−0.050	−8			−0.215 *	−1.12
b_14_				−0.054	6	−3.26		−0.370 *	−2.21 *
b_24_						−2.82		−0.186 *	
b_34_		4.1						0.142	
R^2^_adj_	0.174	0.793	0.896	0.654	0.780	0.415	0.839	0.863	0.734
R^2^_pred_	0.060	0.686	0.848	0.480	0.637	0.179	0.767	0.731	0.480

* p < 0.05, 1 = Fa, 2 = TW, 3 = RS, 4 = PT.

**Table 6:** Panel test results. CV_P*pt*_*,* coefficient of variation of portion mass; DS, difficulty in tablet subdivision; ML*_pt_*, mass loss following splitting into four portions; TB4, percentage of tablets that panelists could break into four portions; TB0, percentage of tablets that panelists could not break.

Batch #	Punch	Ds^•^ Arbitrary Units	TB4 %	TB0 %	ML*_pt_*^•^ %	CV_P*pt*_^□^
1	EBT	1.28 ± 1.72	96.7	0.0	1.15 ± 0.47	11.10 (58)
24	EBT	3.68 ± 2.79	83.3	8.3	0.76 ± 0.31	7.14 (50)
9	TBT	5.05 ± 2.79	71.7	10.0	0.93 ± 0.42	11.74 (43)
21	TBT	9.47 ± 1.28	8.3	81.7	0.61 ± 0.31	4.78 (5)

**^•^** mean ± sd. **^□^** number of tested tablets in brackets.

## Data Availability

Not applicable.
